# Spatial scales of COVID-19 transmission in Mexico

**DOI:** 10.1093/pnasnexus/pgae306

**Published:** 2024-07-31

**Authors:** Brennan Klein, Harrison Hartle, Munik Shrestha, Ana Cecilia Zenteno, David Barros Sierra Cordera, José R Nicolás-Carlock, Ana I Bento, Benjamin M Althouse, Bernardo Gutierrez, Marina Escalera-Zamudio, Arturo Reyes-Sandoval, Oliver G Pybus, Alessandro Vespignani, José Alberto Díaz-Quiñonez, Samuel V Scarpino, Moritz U G Kraemer

**Affiliations:** Network Science Institute, Northeastern University, Boston, MA 02115, USA; Laboratory for the Modeling of Biological & Socio-technical Systems, Northeastern University, Boston, MA 02115, USA; Institute for Experiential AI, Northeastern University, Boston, MA 02115, USA; Network Science Institute, Northeastern University, Boston, MA 02115, USA; Santa Fe Institute, Santa Fe, NM 87501, USA; Network Science Institute, Northeastern University, Boston, MA 02115, USA; Healthcare Systems Engineering, Massachusetts General Hospital, Boston, MA 02114, USA; Prestaciones Económicas y Sociales, Instituto Mexicano del Seguro Social, Ciudad de México, 06600, México; Instituto de Física, Universidad Nacional Autónoma de México, Ciudad de México, 04510, México; Department of Public and Ecosystem Health, College of Veterinary Medicine, Cornell University, Ithaca, NY 14853, USA; Information School, University of Washington, Seattle, WA 98105, USA; Department of Biology, New Mexico State University, Las Cruces, NM 88003, USA; Department of Biology, University of Oxford, Oxford OX1 3SZ, United Kingdom; Colegio de Ciencias Biológicas y Ambientales, Universidad San Francisco de Quito USFQ, Quito 170136, Ecuador; Consorcio Mexicano de Vigilancia Genómica (CoViGen-Mex), Consejo Nacional de Ciencia y Tecnología, Ciudad de México, 03940, México; Pandemic Sciences Institute, University of Oxford, Oxford OX3 7BN, United Kingdom; Department of Biology, University of Oxford, Oxford OX1 3SZ, United Kingdom; Consorcio Mexicano de Vigilancia Genómica (CoViGen-Mex), Consejo Nacional de Ciencia y Tecnología, Ciudad de México, 03940, México; The Jenner Institute, University of Oxford, Oxford OX3 7DQ, United Kingdom; Instituto Politécnico Nacional, IPN, Ciudad de México, 07738, México; Department of Biology, University of Oxford, Oxford OX1 3SZ, United Kingdom; Pandemic Sciences Institute, University of Oxford, Oxford OX3 7BN, United Kingdom; Department of Pathobiology and Population Science, Royal Veterinary College, London AL9 7TA, United Kingdom; Network Science Institute, Northeastern University, Boston, MA 02115, USA; Laboratory for the Modeling of Biological & Socio-technical Systems, Northeastern University, Boston, MA 02115, USA; Health Emergencies Department, Pan American Health Organization, Washington, DC 20037, USA; Instituto de Ciencias de la Salud, Universidad Autónoma del Estado de Hidalgo, Pachuca Hgo, 42160, México; Network Science Institute, Northeastern University, Boston, MA 02115, USA; Institute for Experiential AI, Northeastern University, Boston, MA 02115, USA; Santa Fe Institute, Santa Fe, NM 87501, USA; Department of Biology, University of Oxford, Oxford OX1 3SZ, United Kingdom; Pandemic Sciences Institute, University of Oxford, Oxford OX3 7BN, United Kingdom

**Keywords:** SARS-CoV-2, mobility data, epidemiology, network science, spatial analysis

## Abstract

During outbreaks of emerging infectious diseases, internationally connected cities often experience large and early outbreaks, while rural regions follow after some delay. This hierarchical structure of disease spread is influenced primarily by the multiscale structure of human mobility. However, during the COVID-19 epidemic, public health responses typically did not take into consideration the explicit spatial structure of human mobility when designing nonpharmaceutical interventions (NPIs). NPIs were applied primarily at national or regional scales. Here, we use weekly anonymized and aggregated human mobility data and spatially highly resolved data on COVID-19 cases at the municipality level in Mexico to investigate how behavioral changes in response to the pandemic have altered the spatial scales of transmission and interventions during its first wave (March–June 2020). We find that the epidemic dynamics in Mexico were initially driven by exports of COVID-19 cases from Mexico State and Mexico City, where early outbreaks occurred. The mobility network shifted after the implementation of interventions in late March 2020, and the mobility network communities became more disjointed while epidemics in these communities became increasingly synchronized. Our results provide dynamic insights into how to use network science and epidemiological modeling to inform the spatial scale at which interventions are most impactful in mitigating the spread of COVID-19 and infectious diseases in general.

**Significance Statement**

Outbreaks of infectious diseases, including COVID-19 across different localities are linked via human mobility. Using aggregated human mobility data at the municipality level in Mexico, we find that scales of human mixing and predictability of COVID-19 growth rates shift during the pandemic which improves the ability to target spatial interventions. These dynamical insights are useful when preparing for new outbreaks and planning disease surveillance using a combination of digital mobility and epidemiological data.

## Introduction

The transmission of infectious diseases is highly heterogeneous. Differences in population structure, the landscape of immunity, and environmental factors, result in differences in the timing of outbreaks, their magnitude, and duration (1–15). For infectious diseases, one principal component determining the spatial structure of outbreaks is the frequency of interactions between susceptible and infectious individuals within and between regions (6, 9, 16–19). In most geographies, public health decision-making authority follows political/administrative boundaries (20–26). However, from an epidemiological perspective, the relevant spatial units may not strictly follow political boundaries but rather human mixing (1, 3, 27). Evaluating the spatial structure of COVID-19 transmission remains important in determining optimal interventions (nonpharmaceutical and/or vaccination) to reduce transmission and limit the risk of resurgence of cases (28–31). Whereas prior work has been focused on determining the synchrony of epidemics across spatial units (32–34) we extend that work by investigating how epidemic synchrony varies by mobility informed spatial aggregations.

**Fig. 1.**
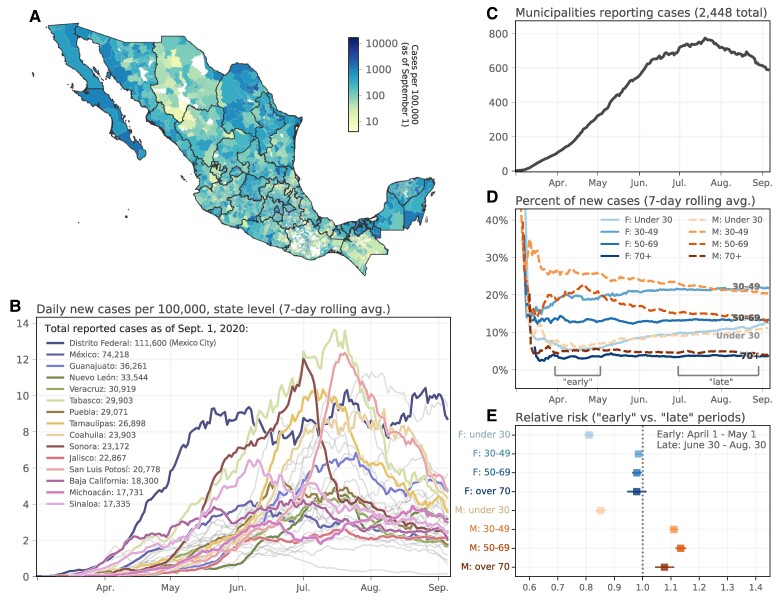
Epidemiological situation of COVID-19 in Mexico. A) Map of cumulative cases per 100,000 people, as of 2020 September 1. B) Timeline of new cases per 100,000 population at the state level (7-day rolling average), highlighting the 15 states with the most severe cumulative outbreaks. C) Number of municipalities that reported confirmed cases of COVID-19 through time. D) Age and sex distributions of confirmed COVID-19 cases across Mexico, highlighting “early” and “late” periods during which the relative risk of infections were calculated. E) Age and sex relative risk ratios of infection, comparing the early vs. late periods from (D).

During the first half of 2020, Mexico experienced one of the largest SARS-CoV-2 epidemics worldwide, with more than 600,000 cases (Fig. 1A,B) and 65,000 confirmed deaths reported between February and September 2020 (35). The epidemic wave peaked in late May in Mexico City (Ciudad de Mexico and formerly known as Distrito Federal) and later ignited epidemics in all other states (36), peaking between June and late July 2020 (Fig. 1B). Mexico has a complex human mobility network with Mexico City playing a pivotal role in determining the dynamics of respiratory infections (37–40). Here, we combine municipality level epidemiological data with weekly anonymized aggregated human mobility data at the same scale, to characterize the spatial scales of the Mexican COVID-19 pandemic and their implications for the implementation of spatially targeted interventions. We investigate whether grouping municipalities by their mobility patterns as opposed to administrative units yields more meaningful epidemiological predictions.

## Materials and methods

### Epidemiological data

Epidemiological data include individual-level information on patients with confirmed RTq-PCR COVID-19 infection between 2020 March–September 30th. Data were downloaded from http://datosabiertos.salud.gob.mx/gobmx/salud/datos_abiertos/datos_abiertos_covid19.zip (last accessed 2020 October 24). Data include information about patients demographics (age and sex) and municipality of residence. In all analyses, we used the date of onset of symptoms.

### Population and travel data

Human mobility and population data were extracted at the municipality level based on the 2016 boundaries (INEGI 2016: https://www.inegi.org.mx/app/mapa/espacioydatos/default.aspx). Population data were downloaded from the COVID-19 indicator dataset, which was provided by INEGI (https://www.inegi.org.mx/investigacion/covid/).

### Aggregated and anonymized human mobility data

We used the Google COVID-19 Aggregated Mobility Research Dataset described in detail in Refs. (41, 42), which contains anonymized relative mobility flows aggregated over users who have turned on the *Location History* setting, which is turned off by default. This is similar to the data used to show how busy certain types of places are in Google Maps—helping identify when a local business tends to be the most crowded. The mobility flux is aggregated per week, between pairs of approximately 5 km^2^ cells worldwide, and for the purpose of this study further aggregated for municipalities in Mexico.

To produce this dataset, machine learning is applied to log data to automatically segment it into semantic trips. To provide strong privacy guarantees (43), all trips were anonymized and aggregated using a differentially private mechanism to aggregate flows over time (see https://policies.google.com/technologies/anonymization). This research is done on the resulting heavily aggregated and differentially private data. No individual user data was ever manually inspected, only heavily aggregated flows of large populations were handled. All anonymized trips are processed in aggregate to extract their origin and destination location and time. For example, if *n* users traveled from location *a* to location *b* within time interval *t*, the corresponding cell (a,b,t) in the tensor would be n±err, where *err* is Laplacian noise. The automated Laplace mechanism adds random noise drawn from a zero mean Laplacian distribution and yields (ϵ,δ)-differential privacy guarantee of ϵ=0.66 and $\delta =2.1\times 10^{29}$ per metric. Specifically, for each week *W* and each location pair (A,B), we compute the number of unique users who took a trip from location *A* to location *B* during week *W*. To each of these metrics, we add Laplace noise from a zero-mean distribution of scale 1/0.66. We then remove all metrics for which the noisy number of users is lower than 100, following the process described in Ref. (43), and publish the rest. This yields that each metric we publish satisfies (ϵ,δ)-differential privacy with values defined above. The parameter ϵ controls the noise intensity in terms of its variance, while *δ* represents the deviation from pure ϵ-privacy. The closer they are to zero, the stronger the privacy guarantees.

These results should be interpreted in light of several important limitations. First, the Google mobility data are limited to smartphone users who have opted into Google’s *Location History* feature, which is off by default. These data may not be representative of the population as whole, and furthermore their representativeness may vary by location. Importantly, these limited data are only viewed through the lens of differential privacy algorithms, specifically designed to protect user anonymity and obscure fine detail. Moreover, comparisons across rather than within locations are only descriptive since these regions can differ in substantial ways.

### Timeline of interventions

The Mexican government has outlined four principle objectives for the control of COVID-19 (i) reduce risk of acquiring infection, (ii) reduce risk of severe morbidity and mortality, (iii) reduce risk and impact on society, and (iv) reduce risk of transmission between infectious and susceptible individuals. We collated a full list of interventions between February and September 2020 and details are provided in Table S1, including references.

### Relative risk model

Following Goldstein and Lipsitch (44), we used age stratified epidemiological data to assess the temporal shifts in the share of a given age group among all cases of infection. To do so, we use the relative risk (RR) (45, 46) statistic that estimates the ratio of the proportion of a given age group among all detected cases of COVID-19 for a later time period vs. an early time period. We selected the early time period to be the month of April (the period right after the implementation of the lockdown) and the late period to be June to September. We adopted the code and model from Goldstein and Lipsitch described in detail (44).

### Community detection algorithm

Human mobility networks, based on data from mobile devices, can be used to capture important population-level trends. Microscopic descriptions often remain too complex to extract meaningful information to describe the transmission process accurately (32). We here use a community detection algorithm following (47) to identify human movement communities (basins) where within-community mobility among municipalities is higher than across-community mobility. We chose this community detection algorithm as it is conceptually related to spatial infectious disease transmission models.

### Municipality-level case growth rates

To estimate the daily epidemic growth rates in each municipality, we fit a mixed effects generalized linear model (GLM) of natural log new daily case counts in sliding 7-day windows (fixed effect; approximately the generation time of COVID-19 in the earliest wave) and a random effect for each municipality on the slope and intercept separately for each municipality, using the R package lme4 v.1.1-21 (48). Daily case counts were determined using the date of symptom onset. Below is the functional form of this regression model for a single municipality. We iterate over each municipality separately and store the resulting growth rate.


(1) $$\begin{array}{rl} {\text{cases}}_{i} & ∼N\left(\alpha_{\;j[i]}+\beta_{1j[i]}(\text{week}),\sigma^{2}\right) \end{array}$$

(2) $$\left(\begin{array}{c} \alpha_{j}\\ \beta_{1j} \end{array}\right)∼N\left(\left(\begin{array}{c} \mu_{\alpha_{j}}\\ \mu_{\beta_{1j}} \end{array}\right),\left(\begin{array}{cc} \sigma_{\alpha_{j}}^{2} & \rho_{\alpha_{j}\beta_{1j}}\\ \rho_{\beta_{1j}\alpha_{j}} & \sigma_{\beta_{1j}}^{2} \end{array}\right)\right).$$

for municipality j=1,…,J

### Relationship between case growth rates and mobility

To test for an effect of mobility from Mexico City on municipality growth rates, we fit a mixed effect GLM with log mobility between Mexico city and each municipality as a fixed effect, a random effect on the intercept for each municipality and a random effect on the slope and intercept for the log mobility each week. The conditional and marginal coefficient of determination, i.e. $R^{2}$, were calculated using the R package MuMIn v1.471. (49) which implements the method developed by Nakagawa et al. (50). Model selection was performed using analysis of variance for mixed effects models as implemented in the R package lmerTest v.3.1-3 (51).


(3) $$\begin{array}{rl} {\text{rate}}_{i} & ∼N\left(\alpha_{\;j[i],k[i]},\sigma^{2}\right) \end{array}$$

(4) $$\begin{array}{rl} & \alpha_{j}∼N\left(\gamma_{0}^{\alpha }+\gamma_{1}^{\alpha }(\text{log 10}(\text{mobility})),\sigma_{\alpha_{j}}^{2}\right),\\ & \;\text{for municipality j}=1,\ldots ,\text{J} \end{array}$$

(5) $$\begin{array}{rl} & \left(\begin{array}{c} \alpha_{k}\\ \gamma_{1k} \end{array}\right)∼N\left(\left(\begin{array}{c} \mu_{\alpha_{k}}\\ \mu_{\gamma_{1k}} \end{array}\right),\left(\begin{array}{cc} \sigma_{\alpha_{k}}^{2} & \rho_{\alpha_{k}\gamma_{1k}}\\ \rho_{\gamma_{1k}\alpha_{k}} & \sigma_{\gamma_{1k}}^{2} \end{array}\right)\right).\\ & \;\text{for week k}=1,\ldots ,\text{K} \end{array}$$

## Results

### Spatial expansion of COVID-19 in Mexico

In Mexico, the spatial range of transmission expanded rapidly after reports of the earliest cases in March 2020, with over 700 municipalities reporting transmission by July 2020 (out of 2,448, Fig. 1C). During April and May, the risk of positive RTq-PCR confirmed cases amongst men aged 30–69 was 1.4 times higher than between July 1 and September 1 (Fig. 1D,E), indicating that the epidemic spread initially within and through these age groups (Fig. S2). This dynamic trend in the demographics of cases is similar to that observed in other countries during the early stages of the pandemic (53, 54).

**Fig. 2.**
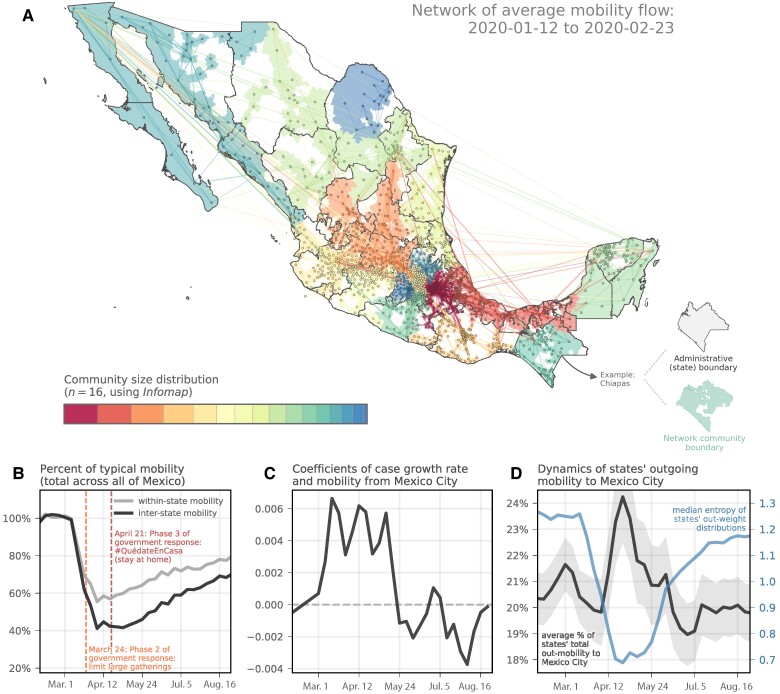
Human mobility and transmission of COVID-19 in Mexico. A) Prepandemic average of the inter-municipality mobility network, colored by network community (detected using the Infomap algorithm). Mobility flow data are based on the aggregated Google Mobility Research dataset (see Materials and methods). B) Deviation of weekly human mobility (number of flows within (grey line) and between states (black line)) from baseline (baseline mobility is calculated as the mean weekly mobility between 2020 January 12 and February 29). C) Evolution of the coefficients of mobility flow from Mexico City in (lagged) correlations with state-level case rates across the country, highlighting the key role that mobility from Mexico City played in the early stage of the epidemic. D) Average fraction of total outgoing mobility from each state that is to Mexico City (black) and the median entropy of states’ distributions of outgoing mobility. Error bands correspond to 95% confidence intervals.

Mexico City experienced early and widespread cases of COVID-19 (Fig. 1B) (36) and due to its centrality including with its surrounding state, Mexico State connecting people from abroad (international arrivals) and within Mexico we hypothesize that human mobility from these two states was a key driver of the spread of COVID-19 in Mexico. Using anonymized, opt-in and aggregated human movement data from mobile phones (see Materials and methods for a more detailed description of the mobility data) we find that case growth rates across Mexican states were associated with human movements from the State of Mexico and Mexico City between March and May 2020 (regression coefficient from generalized linear mixed effects model (fixed effect of municipality and log10 mobility between Mexico City and each municipality; random effects accounting for effects of time and municipality on the slope and intercept) shown in Fig. 2C, conditional $R^{2}=0.62$; see Materials and methods for a detailed description of the statistical model). This pattern is analogous to outbreaks which were driven by major cities in the United Kingdom and China (53, 55). Further, we observe that the share of overall relative human mobility to and from Mexico and Mexico City increased markedly during that period (Fig. 2D) when overall human mobility between states declined (Figs. 2B and S3 showing state-level data on change in human mobility). This points towards a change in the network structure of human mobility in Mexico, as documented in some other countries (56, 57). Overall transmission, and the importance of Mexico City driving the epidemic, declined after the implementation of NPIs through May 2020. However, after the lifting of physical distancing measures on June 1st (see table of documented changes in NPIs, Table S1), case growth rates in the country increased again as a function of mobility from Mexico City, in line with models predicting that lifting lockdowns can lead to reseeding of transmission chains from larger to smaller cities where epidemics were successfully controlled (Fig. 2B, Table S1, (13)).

Variation in weekly new cases within each state in Mexico are generally well predicted by cases in Mexico City weighted by human mobility except for Baja California, Morelos, Chihuahua, Oaxaca, and Chiapas (Fig. S4). We hypothesize that epidemics there were possibly seeded from other countries (United States and Guatemala); further SARS-CoV-2 genomic analyses of unbiased collections of samples will be needed to confirm the SARS-CoV-2 lineage dynamics in these states (34, 36, 55, 58–60). Human mobility data showing cross border (United States to Mexico) movements indicate higher overall mobility to bordering states in Mexico and growth rates in United States–Mexico border states appear higher in the period between May 24–June 28, 2020 (Figs. S5, S6, and S7). The high degree of mobility during that phase resulted in larger case numbers and earlier peaks in states bordering the United States when compared to other states in Mexico (Fig. S6).

### The scales of COVID-19 transmission

It is well known that reductions in mobility (a proxy for reductions in population mixing) have reduced the transmission of COVID-19 within a location especially during the early phase of a disease outbreak (61, 62). However, it remains unclear how structural changes to the mobility network (shifts in the frequency and intensity of mobility within and among regions) have impacted COVID-19 dynamics empirically (56, 57, 63–65). Our underlying hypothesis is that more tightly connected communities exhibit more synchronized epidemic dynamics and, conversely, that more disjointed individual communities have less synchronized epidemics and their epidemics are more likely to fade out (16, 18, 19). We here refer to communities as the equivalent to municipalities and synchrony is defined as the similarity among communities in weekly case growth rates (66)). Both processes have critical implications for disease mitigation and eliminations locally, and at a country level (13, 67–71). The Mexican government announced stringent physical distancing policies on 2020 March 30th which resulted in marked changes in the mobility network (Table S1, Fig. 2B).

To quantify the degree to which mobility patterns are structured by geopolitical boundaries, we use a community detection algorithm that groups municipalities based on their movement patterns (47). Specifically, we aim to identify groups of municipalities such that movements between municipalities within the same group, i.e. community, are more frequent than movements to other municipalities in other communities. Community detection is often accomplished via modularity maximization (72); however, these approaches neglect information about the flow of mobility through the network. Instead, we leverage the *map equation* via an algorithm called InfoMap (47). The InfoMap algorithm utilizes an information theoretic approach to derive expected connectivity patterns if the observed flows were entirely determined by a random walk process. For this study, InfoMap is ideal because it is conceptually related to infectious disease transmission models, which often also utilize stochastic processes (73).

The aim is to identify municipalities where frequent interactions between individuals occur, such that the detected communities approximate the spatial scales of disease transmission (i.e. communities in which it is assumed that infection spreads via contacts within a relatively homogeneously mixing population (74)). Accounting for spatial heterogeneity is known to be important for assessing strategies for interventions (6), especially in areas that have marked differences in urban and rural areas (75). Using this algorithm, we identify 16 mobility communities before the first cases of COVID-19 were detected in Mexico and consider this as the baseline (Fig. 2A). Community size and organization changed following the announcement of the lockdown (2020 March 23 and 30) in Mexico and communities generally became smaller as compared to the pre-pandemic period (fewer municipalities within each community (Figs. S8 and S9 show the communities for each week during the study period). At the peak of the lockdown, we identified approximately 60 mobility communities (a 4-fold increase from the baseline period).

More specifically, there are two notable shifts in the network following the introduction of NPIs. First, more communities are identified but importantly the size of these communities shrinks disproportionately so that one community expands (Mexico City) and many very small ones emerge (Fig. 2D). Further, as a result of the lockdown human movements across municipalities decline more rapidly than movements within a community with one important exception: Mexico City. There we observe that the ratio of within municipality movements declines at a similar rate than movements across municipalities (Fig. S3) further proving its central importance in the mobility network in Mexico.

**Fig. 3.**
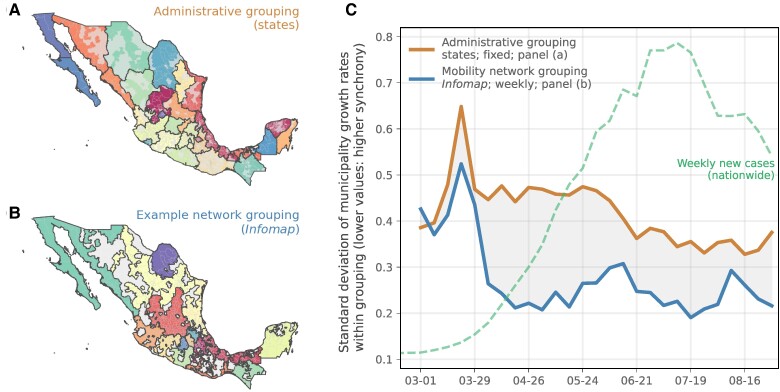
Network structure determines the synchrony of epidemics. A) Grouping of municipalities based on the state administrative boundaries. Gray shaded municipalities are removed from downstream analyses as they could not be assigned a movement community (see Materials and methods). B) Example grouping of municipalities based on human movement data and a community detection algorithm (52) (see Materials and methods). Colors indicate movement communities. Grey municipalities have limited recorded movements and could not be assigned to a community and were consequently excluded from analysis. C) Synchrony of weekly growth rates of epidemics across municipalities as measured by the pairwise standard error between growth rates. The lower the error, the more synchronized epidemics are. Blue line shows grouping by network communities, and orange shows groupings by state administrative boundaries. The green dashed line shows the nationwide trend in reported cases during this period. For comparison, please also see differences in within-state and withing-community standard deviations of growth rates in Fig. S1.

We then compared the weekly infection incidence growth rates within each community and contrasted them to growth rates under a scenario in which municipalities are grouped based on state boundaries (black lines, Fig. 3A,B). As expected, we find that epidemics in municipalities that are grouped by human mobility were more synchronized compared to those grouped by state. (Fig. 3C; see Fig. S1 for a comparison of the within-state and within-community variability in municipality-level epidemic growth rates. Note that the variability is largest in the two states encompassing Mexico City, i.e. México and Distrito Federal, and much smaller in other states. The states encompasing Mexico City are grouped together in community 1, which is reflected in community 1’s much larger observed variability in municipality growth.) The synchrony among municipalities within each community were maximized in April and May 2020, a period when cases were rapidly rising across the country. After June, epidemics that are grouped by movement are still more synchronized, but the differences with groupings by state appear to be smaller (Fig. 3C). This later period (June to October 2020) is a time when Mexico City appears to also lose importance in seeding the epidemic across the country, and local factors (e.g. population size) became more important in determining the epidemic trajectory (76). These results are expected as local factors become more influential in determining disease dynamics (population size, local mixing) and that the importance of continued virus re-importations wanes through time (55).

## Discussion and limitations

We present a generalizable approach for understanding the spatial structure of transmission of COVID-19 and other emerging infectious diseases by accounting for the variations of the human mobility network that occurred as NPIs were implemented in Mexico. We aimed to differentiate the transmission dynamics at a level defined by administrative boundaries from that defined by simple community detection algorithms that are applied to aggregated anonymized weekly human mobility data. We find that as human mobility network structures change, so does the spatial transmission with implications for how interventions might be applied across municipalities identified as having synchronized epidemics. Because most NPIs are implemented around administrative boundaries—even within countries—incorporating these findings into real-world public health decision-making (specifically the coordination of NPIs across highly connected areas) may result in more effective strategies to control an epidemic in Mexico and elsewhere (77–80). The European Commission for example published a report on mobility functional areas (MFAs) which were informed by mobile phone data but the adoption of these recommendations remained sparse (79) and work based on data from the United States in mid-2020 found that a lack of NPI coordination across administrative boundaries could lead to unintended epidemiological consequences (81). Testing our framework beyond Mexico will be important for more coordinated action across countries.

Our model and results are only as accurate as the data that go into them. The Mexican COVID-19 database may suffer from underreporting due to testing shortages, changing case definitions and spatial heterogeneity in reporting (82, 83). For example, relatively few cases were reported from Oaxaca (Fig. 1A) which may be due to barriers to access to testing (84). Additionally, variable access to testing can influence observed epidemic growth rates, which is difficult to control for but unlikely to systematically bias our results on community structure (85, 86). Future extensions of the model and as the pandemic continues will need to take into account high-resolution SARS-CoV-2 data on prior immunity to specific variants. Further, our model is based on higher level descriptions of the population (raw case data and population level human movement data) and these do not capture the high contact heterogeneity within each municipality (e.g. demographic heterogeneity and assortative mixing). These heterogeneities have been shown to be important in the clustering of transmission of COVID-19 with implications for targeted control (32). Contact patterns may differ significantly by age group, employment status and other factors not accounted for in this work. We did however observe heterogeneity in the demographic makeup of cases during the earlier phases of the Mexican COVID-19 pandemic.

Further, results should be interpreted in light of important limitations related to the human mobility data. First, the Google mobility data is limited to smartphone users who have opted into Google’s *Location History* feature, which is off by default. These data may not be representative of the population as whole, and furthermore their representativeness may vary by municipality. Importantly, these limited data are only viewed through the lens of differential privacy algorithms, specifically designed to protect user anonymity and obscure fine detail. Additionally, we used the most common form of the community detection algorithm InfoMap which might not be perfectly suited to represent human mobility patterns. Future work should be focused on developing and testing community detection algorithms that are tailored to the study of epidemics across scales (87–91).

Mexico is composed of 31 free and sovereign states and Mexico City, united under a federation. This means that each administrative region or state is governed by its own constitution, although they are not completely independent of the federal jurisdiction. Furthermore, each state is divided into municipalities, the nation’s basic administrative unit, which possesses limited autonomy (discretionary power on how best to respond to, or apply a public policy). Under a serious nationwide health threat or emergency, such as a pandemic, the federal Ministry of Health (MoH) acquires full authority over the health policies to be implemented nationwide. Nevertheless, Mexican law establishes that the General Health Council (GHC), a collegial body that reports to the president of the republic has the character of health authority, and can emit obligatory norms to be abided by the MoH. The GHC is presided by the Minister of Health and is conformed by federal institutions (e.g. Economy, Communication and Transport) as well as academic institutions, representatives from pharmaceutical industry, and other health system actors (92). Given its mandate and position in the Mexican health system, the GHC constitutes a promising agent to drive public policy outside of the margins or across geo-administrative units. Furthermore, there are examples of inter-state and inter-municipality coordination to resolve problems that extend beyond their borders such as waste management, tax, policing, and perhaps most relevant, health provision. It is in these contexts where evidence-based interventions on innovative approaches, such as the ones presented here become not only an option but a possibility, with greater impact in reducing transmission as compared to approaches where interventions are based on administrative boundaries.

However, theory often differs from practice and reality brings along additional and expected factors into play (e.g. economic (93) and political interests) many of which are not accounted for in this work. Some state governors for example refused to comply with federal health policies in the early relaxation phase in May 2020 (94). Future work focusing on the complex interplay between epidemiological vs. other considerations is important in translating our approach into public health policy.

Mexico has suffered a large and devastating epidemic, and we hope that our findings contribute to a more rational implementation of interventions in the future that can account for the substantial and changing spatial heterogeneity in transmission. Such analyses can be updated and translated to any other country in the world for which aggregated human mobility data are available. Future work should also focus on validating the inferred spatial scales with genomic data (55, 60, 95) or other coarse-graining techniques (96, 97). Developing interventions using patterns observed in empirical mobility networks must be added to the list of priorities for pandemic response and preparedness in the 21st century.

## Supplementary Material

pgae306_Supplementary_Data

## Data Availability

The Google COVID-19 Aggregated Mobility Research Dataset used for this study is available with permission from Google LLC. Publicly available data and all code necessary to recreate the study are hosted on GitHub https://github.com/Emergent-Epidemics/COVID_Mexico and archived on Zenodo https://zenodo.org/doi/10.5281/zenodo.11372046.

## Preprints

A preprint of this article has been published online at https://arxiv.org/abs/2301.13256.
