# Supplementary material for: Spatial scales of COVID-19 transmission in Mexico
Source: PNAS Nexus. 2024 Jul 31;3(9):pgae306. doi: 10.1093/pnasnexus/pgae306 (PMC11404565; doi:10.1093/pnasnexus/pgae306)
Supplement: pgae306_Supplementary_Data [file pgae306_supplementary_data.pdf]

Supplement - Spatial scales of COVID-19 transmission in Mexico

Brennan Klein*[1-3], Harrison Hartle[1,4], Munik Shrestha[1], Ana Cecilia Zenteno[5], David Barros Sierra Cordera[6], José R. Nicolas-Carlock[7], Ana I. Bento[8], Benjamin M. Althouse[9,10], Bernardo Gutierrez[11-13], Marina Escalera-Zamudio[11,13], Arturo Reyes-Sandoval[14,15], Oliver G. Pybus[11,16,18], Alessandro Vespignani[1,2], Jose Alberto Diaz-Quiñonez*†[17], Samuel V. Scarpino*‡[1,3,4], and Moritz U.G. Kraemer*§[11,18]

[1] *Network Science Institute, Northeastern University, Boston, Massachusetts, USA*
[2] *Laboratory for the Modeling of Biological & Socio-technical Systems, Northeastern University, Boston, Massachusetts, USA*
[3] *Institute for Experiential AI, Northeastern University, Boston, Massachusetts, USA*
[4] *Santa Fe Institute, Santa Fe, New Mexico, USA*
[5] *Massachusetts General Hospital, Boston, Massachusetts, USA*
[6] *Instituto Mexicano del Seguro Social, Ciudad de México, México*
[7] *Instituto de Investigaciones Jurídicas, Universidad Nacional Autónoma de México, Ciudad de México, México*
[8] *Department of Epidemiology and Biostatistics, School of Public Health, Indiana University, Bloomington, Indiana, USA*
[9] *Information School, University of Washington, Seattle, Washington, USA*
[10] *Department of Biology, New Mexico State University, Las Cruces, New Mexico, USA*
[11] *Department of Biology, University of Oxford, Oxford, UK*
[12] *School of Biological & Environmental Sciences, Universidad San Francisco de Quito, Quito, Ecuador*
[13] *Consorcio Mexicano de Vigilancia Genómica*
[14] *The Jenner Institute, University of Oxford, Oxford, UK*
[15] *Instituto Politécnico Nacional, IPN, Ciudad de México, México*
[16] *Department of Pathobiology and Population Science, Royal Veterinary College, London, UK*
[17] *Instituto de Ciencias de la Salud, Universidad Autónoma del Estado de Hidalgo, Pachuca, Hidalgo, México*
[18] *Pandemic Sciences Institute, University of Oxford, UK*

*b.klein@northeastern.edu
†alberto_diaz@uaeh.edu.mx
‡s.scarpino@northeastern.edu
§moritz.kraemer@biology.ox.ac.uk

A Extended Data Figures

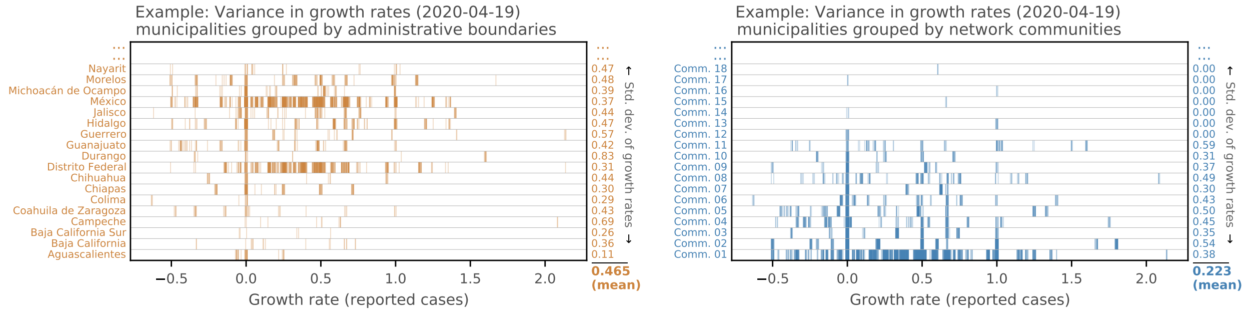

Figure S.1: For one example week (April 19, 2020), comparison of the mean standard deviations in municipality growth rates within either (left) administrative (states) boundaries or (right) network communities. In each panel, the standard deviations of municipalities' infection growth rates within each grouping (state vs. network community) is shown on the right. Figure 3c shows the average of these values over time.

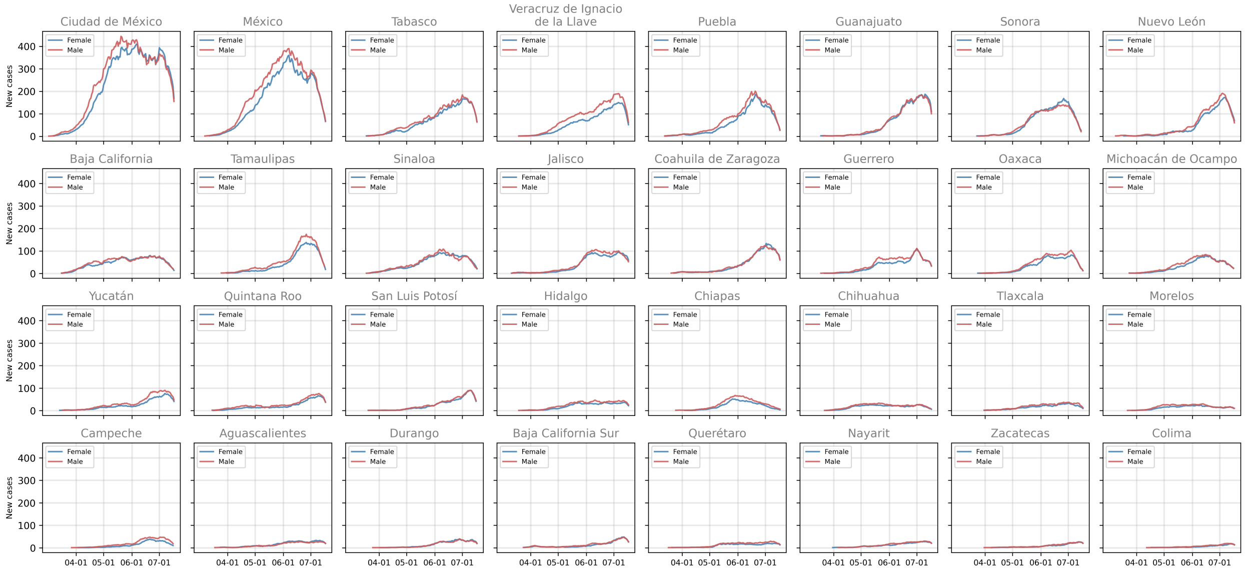

Figure S.2: Number of new cases per state and sex (7-day average).

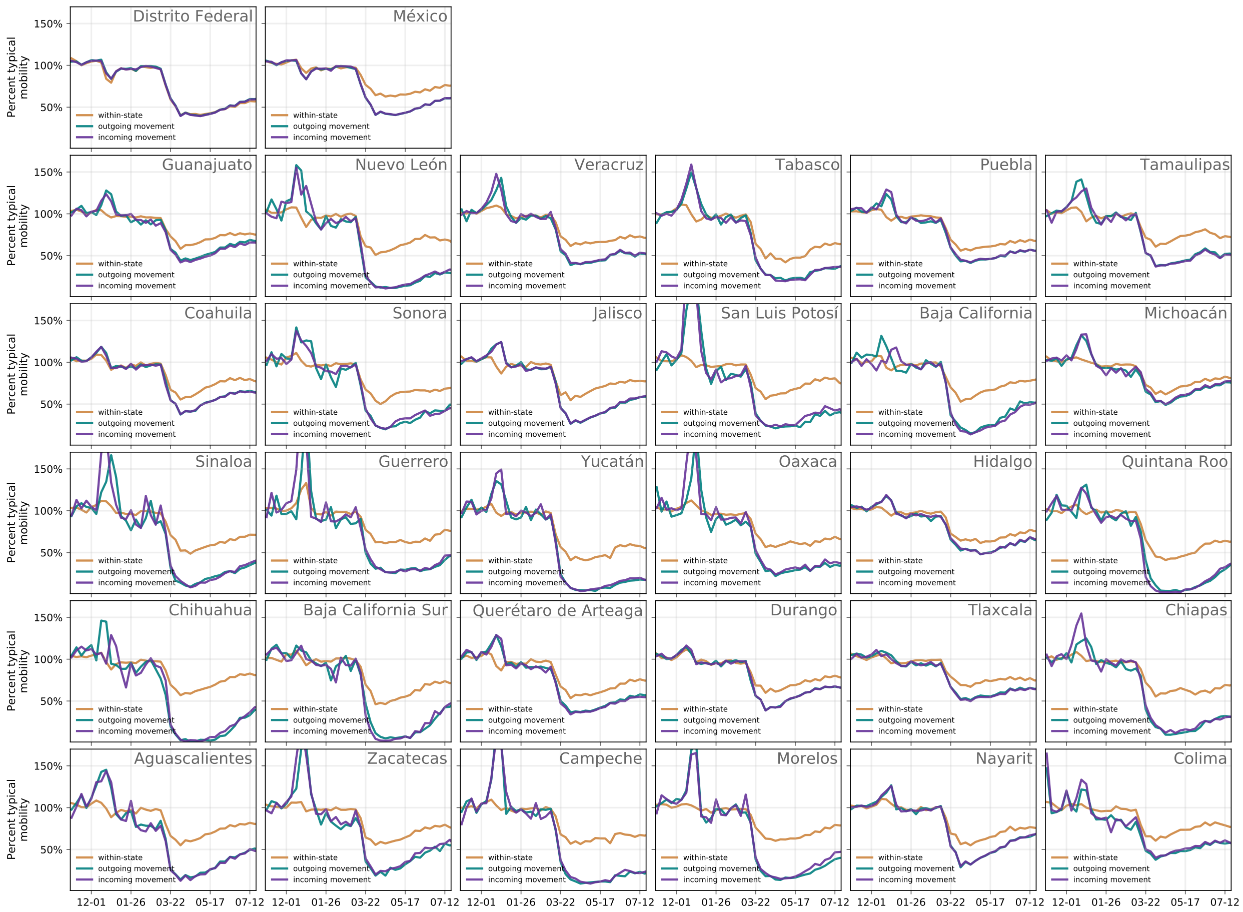

Figure S.3: Weekly relative change in human mobility within each state and between states (incoming and outgoing) as compared to baseline.

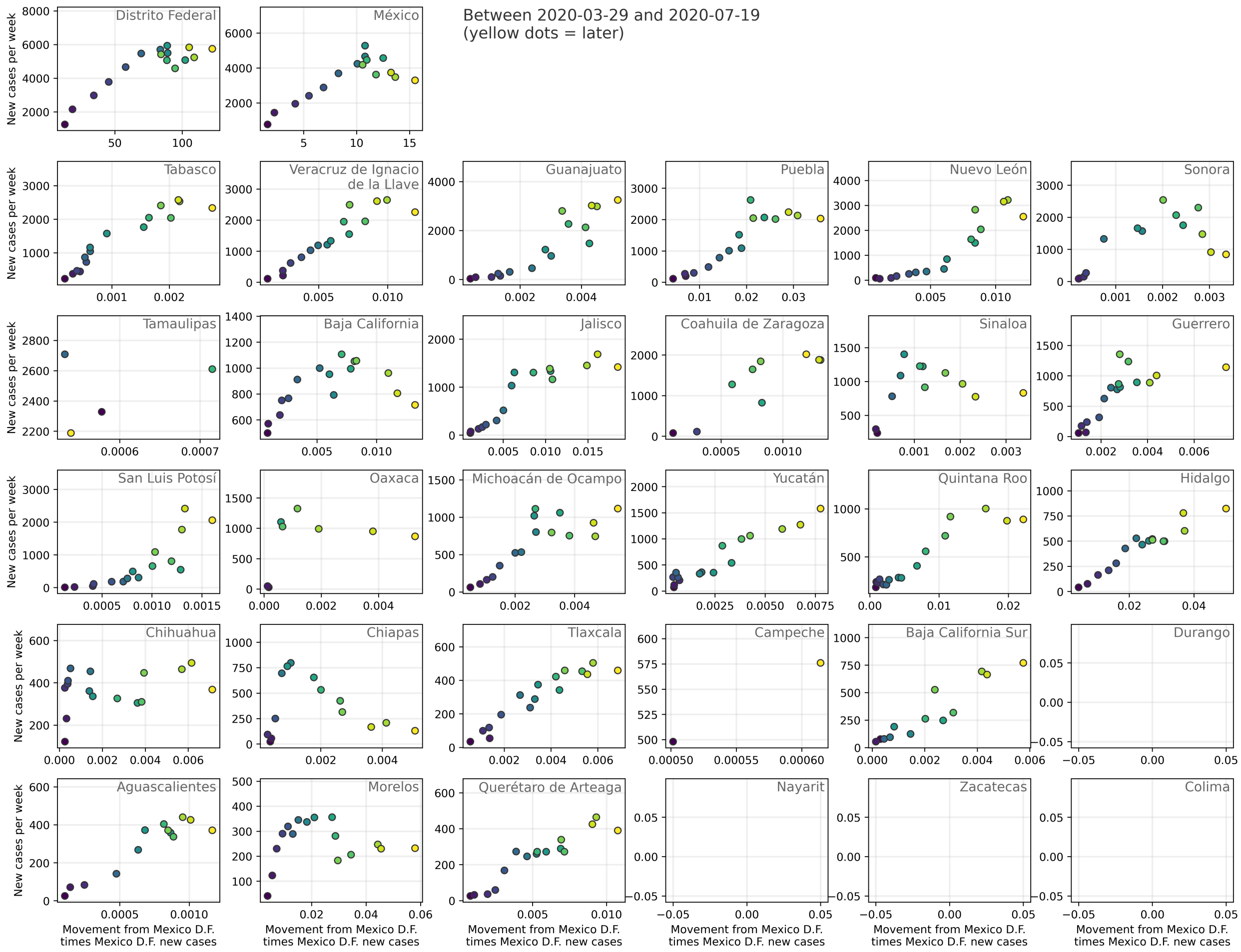

Figure S.4: State-specific correlations of new reported cases (weekly) vs. mobility from Mexico City *times* new reported cases in Mexico City (weekly). States with low mobility and case count data coverage are included but not plotted in this figure.

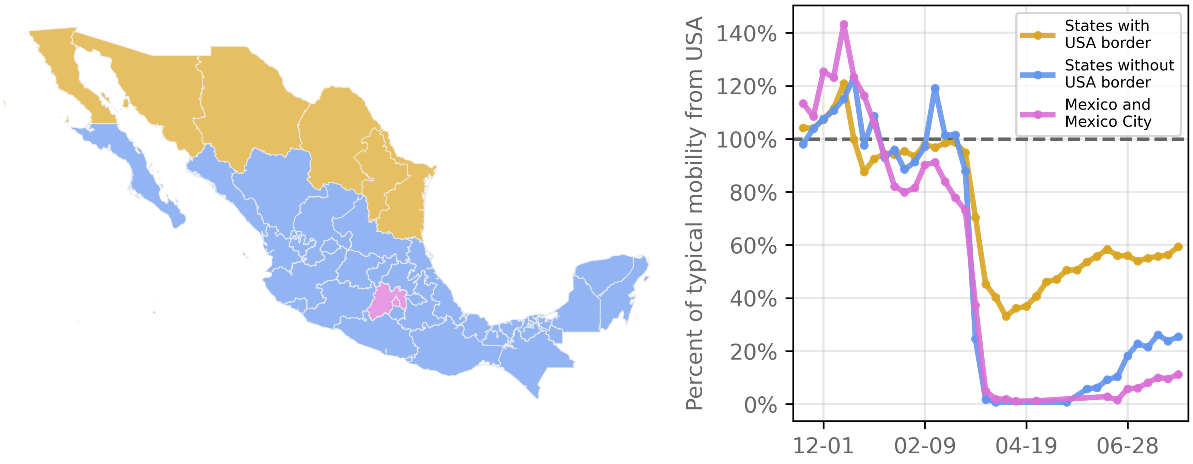

Figure S.5: Weekly relative human mobility where the origin is the USA and the destination are states in Mexico divided into states that share a land border, Mexico and Mexico City and all other states.

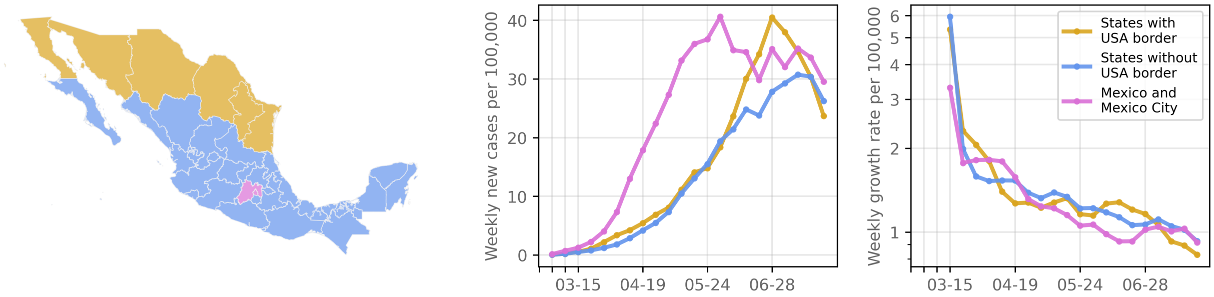

Figure S.6: Weekly new cases per 100,000 divided into cases in Mexico City and the state of Mexico, states that share a land border with the USA, and all other states.

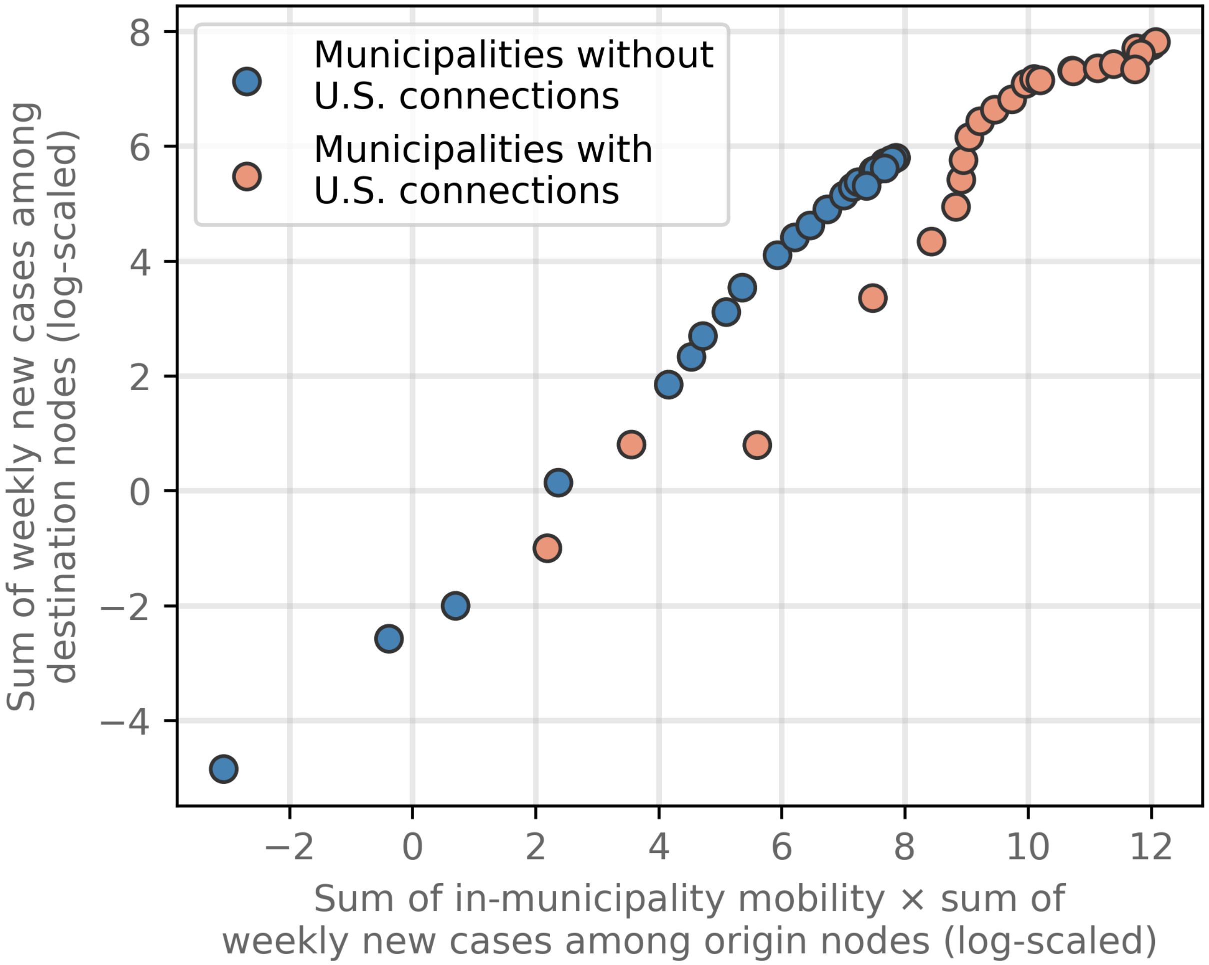

Figure S.7: Weekly number of cases among municipalities in Mexico coloured by their geographic position to the USA (bordering vs. not bordering) and the sum of in-municipality mobility × weekly new cases among origin nodes (both on the log scale).

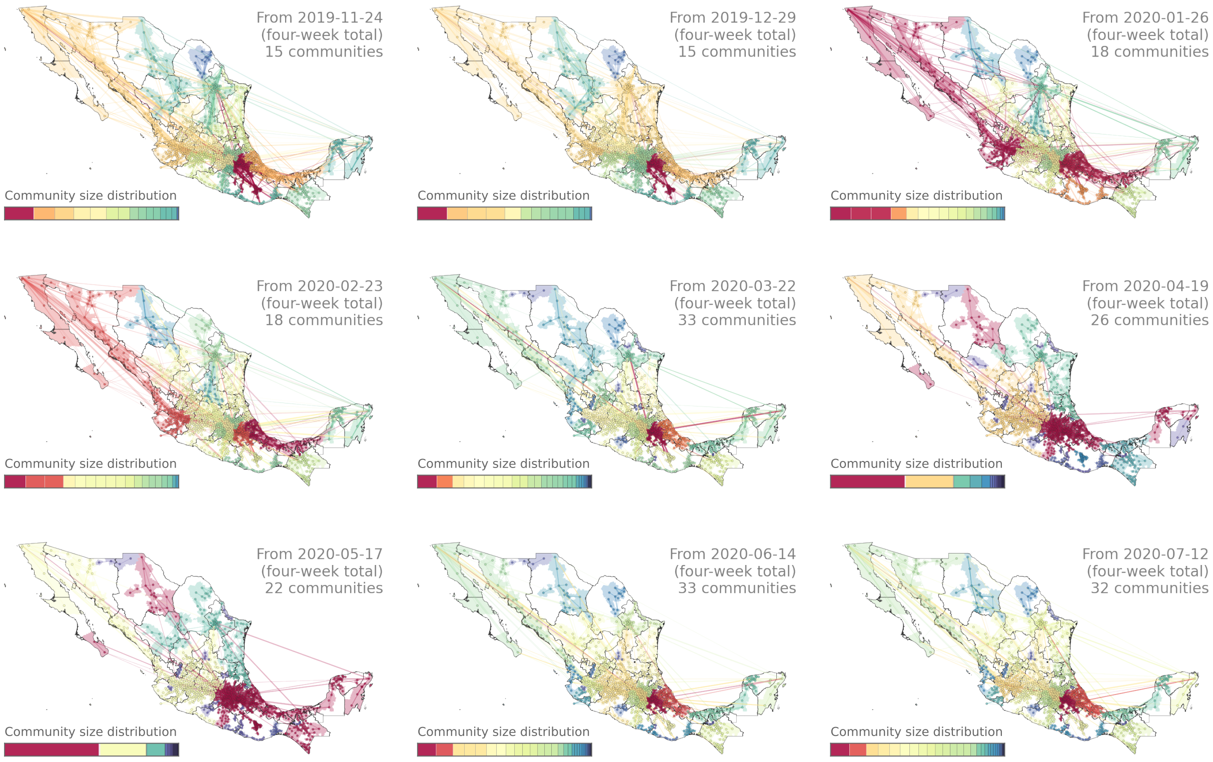

Figure S.8: Four-week snapshots of mobility in Mexico. Weekly human mobility in Mexico at the municipality level. Thickness of lines represents intensity of relative mobility flow. Colours represent the membership to movement communities as estimated using the map equation (Materials & Methods).

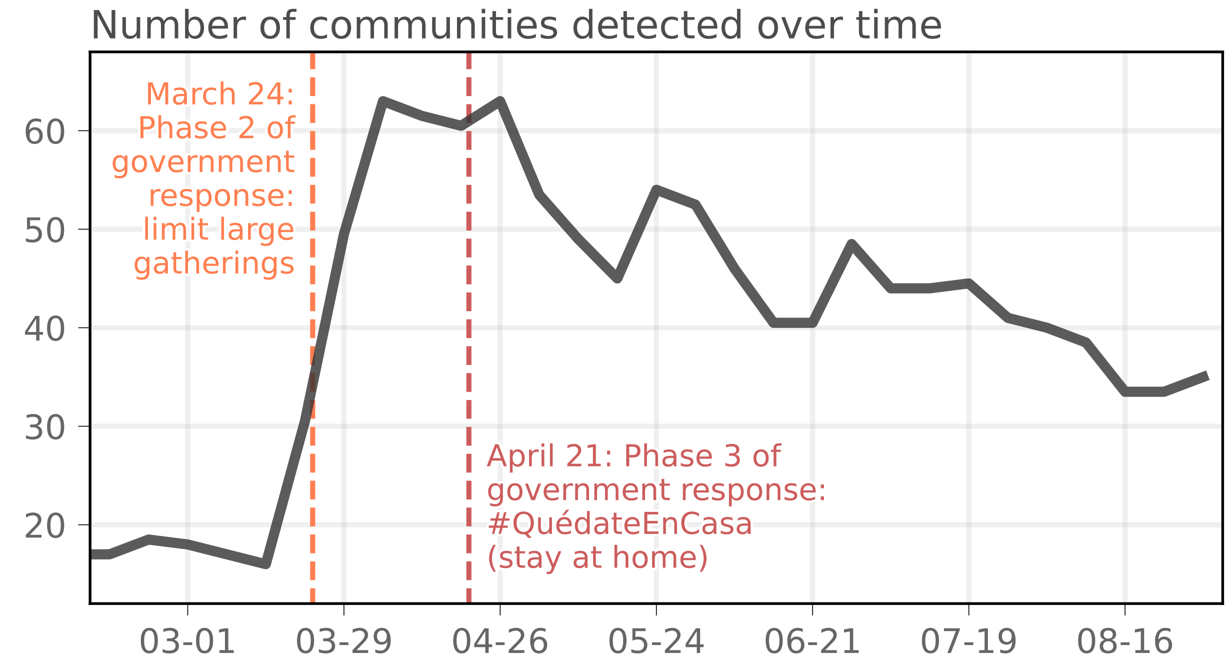

Figure S.9: Number of communities detected each week during the first wave of the COVID-19 epidemic in Mexico.

Date	Intervention
March 16, 2020	Mexican Secretariat of Public Education (SEP) suspend classes in schools of preschool, primary, secondary education, as well as those of the upper middle and higher types dependent on the SEP [1].
March 17, 2020	Universities begin to suspend classes and social events [2].
March 20, 2020	Mexican Secretariat of Public Education (SEP) cancels all civic and sports events [3].
March 21, 2020	United States - Mexico border was closed to non-essential travel but remained open for commerce. Closure extended until November 21, 2020 [4].
March 23/24, 2020	National period of social distancing begins. Schools closed and all non-essential operations were closed including gatherings of 100+ people [5].
March 30, 2020	National health emergency declared. Policies included: (1.) Non-essential services suspended. (2.) Private sector is asked to require employees to work from home. (3.) Sectors that kept operating normally: government, health (public and private), public safety, social programs, critical infrastructure, and essential services. A full list of essential services can be viewed here: https://www.dof.gob.mx/nota_detalle.php?codigo=5590914&fecha=31/03/2020. (4.) People over 60 years old are urged to stay home. (5.) Public gatherings of over 50 people are banned. (6.) There was no enforced curfew. Expiration date: April 30, 2020.
April 5, 2020	Hospital reconversion strategy guidelines published in order to contain nosocomial transmission [6]
April 16, 2020	The Federal Government announces the extension of the health emergency and emphasizes the need to restrict movement to and from areas of high transmissibility until May 30th [7].
May 14, 2020	Ministry of Health announces epidemiologic color-coded system to re-open social, educational, economic activities at state level [8].
May 18, 2020	First phase of the "new normality" 324 municipalities with no recorded COVID-19 cases are given green light to reopen businesses and schools [9]. Car factories were meant to reopen on June 1st, but began reopening on May 18th under US pressure (Factories remained closed from March 23rd, to May 18th).

June 1, 2020	Mexico's national period of social distancing concludes. A new color-coded system was enacted across the country to assess how quickly states can reopen their economies and schools: red, orange, yellow, and green [10].
July 20, 2020	Daycare centers run by the country's social security system reopened in coordination with local authority and based on color-coded indicators. Currently, all but 4 states have daycare centers open. [11]
October 18, 2020	The Health Ministry announced that 17 states—Mexico City included—were at alert level orange and 14 were at yellow. Only one state, Campeche, was at green [12]. In states at the orange level, businesses such as hotels and restaurants can reopen while following health protocols such as enforcing limited capacity. Yellow allows for most economic activities to return to normal with some occupancy limits.
October 22, 2020	Some local governments have chosen to enact more stringent restrictions than the federal government guidelines, e.g., Jalisco and Chihuahua [13].

Table S.1: Timeline of government interventions in Mexico.

A.1 Citation diversity statement

Recent work has quantified bias in citation practices across various scientific fields; namely, women and other minority scientists are often cited at a rate that is not proportional to their contributions to the field [14–21]. In this work, we aim to be proactive about the research we reference in a way that corresponds to the diversity of scholarship in this field. To evaluate gender bias in the references used here, we obtained the gender of the first/last authors of the papers cited here through either 1) the gender pronouns used to refer to them in articles or biographies or 2) if none were available, we used a database of common name-gender combinations across a variety of languages and ethnicities. By this measure (excluding citations to datasets/organizations, citations included in this section, and self-citations to the first/last authors of this manuscript), our references contain 4% woman(first)-woman(last), 24% woman-man, 17% man-woman, 48% man-man, 0% nonbinary, 7% man solo-author, and 0% woman solo-author. This method is limited in that an author's pronouns may not be consistent across time or environment, and no database of common name-gender pairings is complete or fully accurate.

Supplemental References

[1] *DOF - Diario Oficial de la Federación.* URL: https://www.dof.gob.mx/nota_detalle.php?codigo=5589479&fecha=16/03/2020.

[2] *Coronavirus en México: universidades suspenden clases y se intensifican las acciones preventivas.* 2020. URL: https://www.infobae.com/america/mexico/2020/03/13/coronavirus-en-mexico-universidades-suspenden-clases-y-se-intensifican-las-acciones-preventivas/.

[3] *Gobierno de México suspenderá todas las actividades escolares por coronavirus.* 2020. URL: https://www.latimes.com/espanol/mexico/articulo/2020-03-14/gobierno-de-mexico-suspendera-todas-las-actividades-escolares-por-coronavirus.

[4] U.S. Embassy & Consulates in Mexico. *Mexico, U.S.M. to. Travel restrictions - Fact sheet.* 2021. URL: https://mx.usembassy.gov/travel-restrictions-fact-sheet/.

[5] *Inicia fase 2 por coronavirus COVID-19 – Coronavirus.* URL: https://coronavirus.gob.mx/2020/03/24/inicia-fase-2-por-coronavirus-covid-19/.

[6] Gobierno de México and Secretaría de Salud COVID-19. *Lineamiento de Reconversión Hospitalaria.* URL: https://coronavirus.gob.mx/wp-content/uploads/2020/04/Documentos-Lineamientos-Reconversion-Hospitalaria.pdf.

[7] *Coronavirus en México: guía para entender las cuatro nuevas medidas de control y prevención del COVID-19 cercanas a la Fase 3.* URL: https://www.infobae.com/america/mexico/2020/04/16/coronavirus-en-mexico-guia-para-entender-las-cuatro-nuevas-medidas-de-control-y-prevencion-del-covid-19-cercanas-a-la-fase-3/.

[8] *DOF - Diario Oficial de la Federación*. URL: https://dof.gob.mx/nota_detalle.php?codigo=5593313&fecha=14/05/2020#gsc.tab=0.

[9] *Conferencia 16 de mayo – Coronavirus*. URL: https://coronavirus.gob.mx/2020/05/16/conferencia-16-de-mayo-2/.

[10] Subsecretaría de Prevención y Promoción de la Salud. *Semáforo de riesgo epidemiológico: COVID-19: indicadores y metodología*. URL: https://coronavirus.gob.mx/wp-content/uploads/2020/06/Lineamiento_Semaforo_COVID_05Jun2020_1600.pdf.

[11] *IMSS nurseries open on July 20, after supervision of health protocols*. URL: http://www.imss.gob.mx/prensa/archivo/202007/463.

[12] *COVID-19 MÉXICO Comunicado Técnico Diario - 18 Octubre 2022*. URL: http://saludsinaloa.gob.mx/wp-content/uploads/2020/reportescovid/Covid19ReporteDiario18Noviembre2020.pdf.

[13] *State Government publishes new agreement on Red Light measures*. URL: https://chihuahua.gob.mx/contenidos/publica-gobierno-del-estado-nuevo-acuerdo-de-medidas-del-semaforo-rojo.

[14] Perry Zurn, Danielle S. Bassett, and Nicole C. Rust. "The citation diversity statement: A practice of transparency, a way of life". In: *Trends in Cognitive Sciences* 24.9 (2020), pp. 669–672. DOI: 10.1016/j.tics.2020.06.009.

[15] Jordan D. Dworkin, Kristin A. Linn, Erin G. Teich, Perry Zurn, Russell T. Shinohara, and Danielle S. Bassett. "The extent and drivers of gender imbalance in neuroscience reference lists". In: *Nature Neuroscience* 23.8 (2020), pp. 918–926. DOI: 10.1038/s41593-020-0658-y.

[16] Paula Chakravartty, Rachel Kuo, Victoria Grubbs, and Charlton McIlwain. "#CommunicationSoWhite". In: *Journal of Communication* 68.2 (2018), pp. 254–266. DOI: 10.1093/joc/jqy003.

[17] Daniel Maliniak, Ryan Powers, and Barbara F. Walter. *The gender citation gap in international relations*. Vol. 67. 4. 2013, pp. 889–922. DOI: 10.1017/S0020818313000209.

[18] Michelle L. Dion, Jane Lawrence Sumner, and Sara Mc Laughlin Mitchell. "Gendered citation patterns across political science and social science methodology fields". In: *Political Analysis* 26.3 (2018), pp. 312–327. DOI: 10.1017/pan.2018.12.

[19] Neven Caplar, Sandro Tacchella, and Simon Birrer. "Quantitative evaluation of gender bias in astronomical publications from citation counts". In: *Nature Astronomy* 1 (2017). DOI: 10.1038/s41550-017-0141.

[20] Pierre Azoulay and Freda Lynn. "Self-citation, cumulative advantage, and gender inequality in science". In: *Sociological Science* 7 (2020). DOI: 10.15195/v7.a7.

[21] Gita Ghiasi, Philippe Mongeon, Cassidy R. Sugimoto, and Vincent Larivière. "Gender homophily in citations". In: *23rd International Conference on Science and Technology Indicators* (2018), pp. 1519–1525. URL: https://hdl.handle.net/1887/65291.
